# Effects of a DPP4 inhibitor on cisplatin-induced acute kidney injury: study protocol for a randomized controlled trial

**DOI:** 10.1186/s13063-015-0772-4

**Published:** 2015-05-29

**Authors:** Seon Ha Baek, Se Hyun Kim, Jin Won Kim, Yu Jung Kim, Keun-Wook Lee, Ki Young Na

**Affiliations:** Division of Nephrology, Department of Internal Medicine, Seoul National University Bundang Hospital, 82, Gumi-ro 173 Beon-gil, Bundang-gu, Seongnam, Gyeonggi-do 463-707 South Korea; Division of Hematology and Medical Oncology, Department of Internal Medicine, Seoul National University Bundang Hospital, 82, Gumi-ro 173 Beon-gil, Bundang-gu, Seongnam, Gyeonggi-do 463-707 South Korea

**Keywords:** Acute kidney injury, Cisplatin, DPP4 inhibitor, Nephrotoxicity

## Abstract

**Background:**

Cisplatin is a potent chemotherapeutic agent, but its nephrotoxicity, which results in acute kidney injury (AKI), often limits its clinical application. Although many studies have attempted to target the mechanism responsible for its nephrotoxicity, no such method has been demonstrated to be effective in clinical trials. Recently, a dipeptidyl peptidase-4 (DPP4) inhibitor has been reported to have a renoprotective effect in a mouse model of cisplatin-induced AKI. Therefore, we will evaluate whether a DPP4 inhibitor protects the kidney from cisplatin-induced injury in humans.

**Methods/Design:**

This is a single-center, prospective, randomized, double-blind, placebo-controlled trial. A total of 182 participants who are scheduled for cisplatin treatment will be enrolled and randomly assigned to receive either a DPP4 inhibitor (gemigliptin) or a placebo. Participants will take the study drugs for 8 days starting 1 day before cisplatin treatment. The primary outcome of interest is the incidence of AKI at 7 days after finishing treatment with cisplatin. The secondary outcomes include changes in serum creatinine levels and estimated glomerular filtration rates from baseline to 7 days after cisplatin treatment.

**Discussion:**

This is the first clinical trial to investigate the effect of a DPP4 inhibitor on cisplatin-induced AKI.

**Trial registration:**

ClinicalTrials.gov number NCT02250872, December 26, 2014.

## Background

Cisplatin is a potent chemotherapeutic agent that is approved for the treatment of several types of cancer, including bladder cancer, cervical cancer, non-small cell lung cancer, and squamous cell carcinoma of the head and neck [1]. However, the kidneys are particularly affected by cisplatin because it accumulates there [1]. Cisplatin nephrotoxicity is manifested by hypokalemia, hypomagnesemia, and acute kidney injury (AKI), which often limit its clinical use [1–4]. Impairment of renal function is common and dose-dependent. AKI is found in 6–35 % of patients treated with a single dose of cisplatin and in 50–75 % of patients treated with multiple doses [5–7].

Abnormal renal hemodynamics and renal tubular cell toxicity resulting from oxidative stress, apoptosis, and inflammation are considered to be the mechanisms of cisplatin-induced nephrotoxicity [1, 8–14]. Different strategies targeting each mechanism of nephrotoxicity have been evaluated for their abilities to prevent and/or attenuate renal injury [1, 15, 16]. However, none of these strategies has been demonstrated to be effective in clinical trials [15].

Dipeptidyl peptidase-4 (DPP4) inhibitors are currently used in the treatment of type 2 diabetes mellitus to improve glucose tolerance by increasing the half-lives of glucagon-like peptide-1 (GLP-1) and glucose-dependent insulinotropic peptide [17, 18]. However, in addition to the glucose-lowering effects of DPP4 inhibitors, tissue-protective effects of DPP4 inhibition have been also demonstrated [19–23]. In particular, studies have shown that DPP4 inhibitors can protect the kidney from diabetic nephropathy, ischemia-reperfusion injury, and chronic kidney disease [24–28]. Recently, Kataqiri *et al*. have reported that a DPP4 inhibitor has a renoprotective effect in rodent cisplatin-induced AKI models by enhancing GLP-1 signaling [29]. Based on these reports of the effects of DPP4 inhibitors, we hypothesize that treatment with a DPP4 inhibitor will have a beneficial effect in cisplatin-induced AKI.

Therefore, we will conduct this clinical trial in patients treated with cisplatin.

## Methods/design

### Hypothesis

Treatment with a DPP4 inhibitor will prevent and/or ameliorate cisplatin-induced AKI in humans. Compared with placebo-treated patients, the incidence of AKI will be lower in DPP4 inhibitor-treated patients.

### Study design

**Fig. 1:**
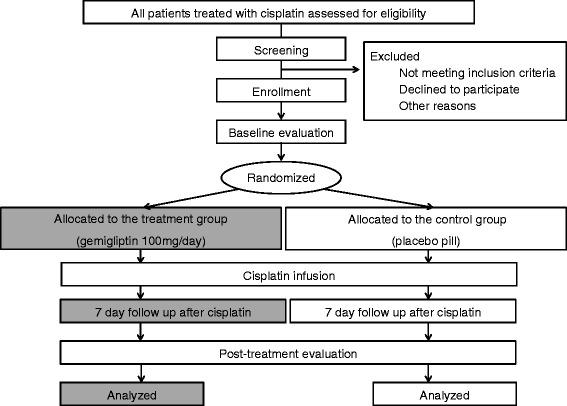
Study algorithm

This is a single-center, prospective, randomized, double-blind, placebo controlled study. This study is an investigator-initiated clinical trial. The study algorithm is described in Fig. 1. After enrollment, clinical follow-up will be performed 7 days after cisplatin treatment.

### Study participants and measurements

Cancer patients aged 18–70 years treated with intravenous cisplatin will be screened. The following will be conducted at the initial visit: (1) a questionnaire regarding active cancer history, chemotherapy, medical history, and history of nephrotoxic use including the use of nonsteroidal anti-inflammatory drugs (NSAIDs), antibiotics, contrast media, and calcineurin inhibitors; (2) a physical examination of all systems; (3) height and weight measurements; (4) blood pressure and pulse rate measurements. Participants who meet all of the inclusion and exclusion criteria and provide written, informed consent are eligible for this study (Table 1).

**Table 1:** Inclusion and exclusion criteria

Inclusion criteria	Exclusion criteria
Age between 18 and 70	Diabetes mellitus
	Chronic kidney disease stage IV-V (eGFR <30 ml/min/1.73m^2^)
	History of transplantation
	History of acute kidney injury before randomization
	Active infection
Cancer patients treated with intravenous cisplatin	Use of other nephrotoxic agents such as nonsteroidal anti-inflammatory drugs, aminoglycosides, colistin, vancomycin, cyclosporine, tacrolimus
	Receiving contrast media during last 72 h
	Liver disease (bilirubin >2 mg/dl, transaminase levels >2.5 times the upper limit normal)
Written consent	Patients with high risks of dehydration owing to poor oral intake
	High blood pressure (>180/110 mmHg despite antihypertensive medications)
	Hypersensitivity to gemigliptin or its excipients
	Low compliance to gemigliptin treatment

Abbreviation: *eGFR* estimated glomerular filtration rate

Serum creatinine (SCr) will be measured by the isotope dilution mass spectrometry-traceable method using a Toshiba TBA 200FR Analyzer (Toshiba, Tokyo, Japan). The estimated glomerular filtration rate (eGFR) will be calculated using the Chronic Kidney Disease Epidemiology Collaboration equations (CKD EPI). The CKD EPI formula, expressed as a single equation, is eGFR = 141 × min (SCr/κ, 1)^α^ × max (SCr/κ, 1)^-1.209^ × 0.993^Age^ × 1.018 [if female] × 1.159 [if black], where κ is 0.7 for females and 0.9 for males, α is −0.329 for females and −0.411 for males, min indicates the minimum of SCr/κ or 1, and max indicates the maximum of SCr/κ or 1 [30] .

### Randomization

A research coordinator will conduct the randomization and deliver the study drug. The participants and investigators will be blinded to the treatment assignment. A list of random numbers will be generated by an independent statistician. Eligible participants will be randomly assigned 1:1 to either the treatment group or the control group in accordance with the predefined randomization list with a block size of four. The randomization will be stratified on the basis of the number of times cisplatin is administered (one or more than two) and on the cisplatin dose (< or ≥50 mg/m^2^) and will utilize a randomized block design.

### Treatments

**Fig. 2:**
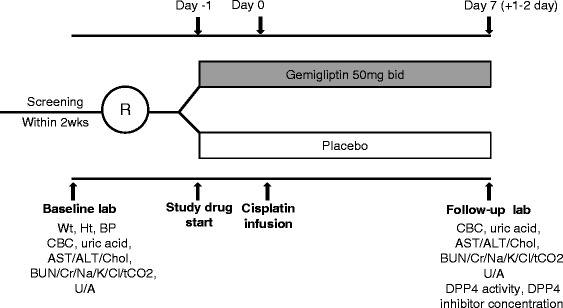
Study schedule. Abbreviations: *R*, randomization; *Wt*, weight; *Ht*, height; *BP*, blood pressure; *CBC*, complete blood count; *AST*, aspartate aminotransferase; *ALT*, alanine aminotransferase; *chol*, cholesterol; *BUN*, blood urea nitrogen; *Cr*, creatinine; *tCO*_*2*_, total CO_2_; *U*/*A*, urinalysis; *DPP4*, dipeptidyl peptidase 4

A selective DPP4 inhibitor, gemigliptin, which is clinically available, will be used in this study. The gemigliptin and placebo tablets will be provided by LG Life Sciences (Seoul, Korea). After randomization, the participants will take either a gemigliptin (treatment group) or placebo (control group) pill for 8 days starting 1 day before cisplatin treatment. The tablet shapes and packaging of the placebo pills are identical to those of the gemigliptin pills. The prescription and administration of the study drugs will be conducted in a double-blind manner. Based on a study that reported a renoprotective effect of a DPP4 inhibitor in a mouse model of cisplatin-induced AKI [29], 100 mg/day of gemigliptin will be administered to the participants in two divided doses for 8 days starting 1 day before cisplatin treatment (Fig. 2). The administration of other nephrotoxic drugs, such as NSAIDs, aminoglycosides, colistin, and vancomycin, will be prohibited during the study, and exposure to contrast media during the 72 h prior to treatment will result in exclusion from the study. Investigators or research coordinators will evaluate drug compliance by counting pills, and participants with less than 80 % compliance will be removed from the study.

### Chemotherapy regimen and precautions

A hydration protocol will be identically applied to both the treatment and control groups. Cisplatin will be mixed with 500 ml of isotonic saline and will be infused into each patient over 30 min. Participants treated with ≥50 mg/m^2^ of cisplatin will receive 2,000 ml of 0.45 % saline and 100 ml of 15 % mannitol. Participants treated with <50 mg/m^2^ of cisplatin will receive 1,000 ml of 0.45 % saline (Table 2).

**Table 2:** Hydration protocol

Cisplatin dose	Before and after cisplatin infusion	During cisplatin infusion
≥50 mg/m^2^	2,000 ml of 0.45 % saline with 100 ml of 15 % mannitol	Mix cisplatin in 500 ml of isotonic saline
<50 mg/m^2^	1,000 ml of 0.45 % saline	Mix cisplatin in 500 ml of isotonic saline

### Outcome measures

The primary outcome of interest is the incidence of AKI at 7 days after cisplatin treatment, where AKI is defined as any of the following: an increase in SCr levels of ≥0.3 mg/dl, an increase in SCr levels of 50 % compared with baseline levels, or a decrease in the eGFR by ≥25 % compared with baseline [31–33]. The secondary outcomes will include changes in SCr levels and the eGFR compared with baseline values for 7 days after cisplatin treatment.

### Clinical and laboratory evaluations

The physical examination and medication reviews will be conducted before and after 8 days of treatment. Laboratory evaluations will be performed twice, before and after 8 days of treatment; the laboratory evaluations will include a complete blood count; measurements of the levels of hemoglobin, electrolytes, SCr, calcium, phosphorous, protein, albumin, and glucose; a liver function test; and urinalysis. DPP4 activity and the concentration of the DPP4 inhibitor will be measured in serum and plasma after treatment.

### Safety issues

Any occurrence of headache, diarrhea, upper respiratory tract infection, hypoglycemia, or hypersensitivity reaction will be recorded during treatment with gemigliptin/placebo. All adverse events, including serious adverse events, will be recorded and followed up during the study period or until resolution. All serious adverse events will be graded and reported to investigators and the ethics committee.

### Sample size calculations

No previous study has evaluated the effect of a DPP4 inhibitor on cisplatin-induced AKI. We expect that the incidence of AKI will be 25 % in patients treated with a single dose of cisplatin and that treatment with a DPP4 inhibitor could reduce this incidence by 60 %. We calculated the required sample size for an estimated dropout rate of 20 %, a one-sided level of significance of α = 5 %, and a power of 80 % and found that 91 participants will be needed in each group to find a significant difference using a *χ*^2^ test. A total of 182 participants will be included in the analysis.

### Statistical analyses

Statistical analyses will be conducted on both per protocol (PP) and intention-to treat (ITT) bases. For the PP analysis, all participants who completed the study will be included to evaluate the primary and secondary outcomes. For the ITT analysis, all participants who were enrolled and randomized to one of the two groups will be included.

The baseline characteristics and laboratory data will be presented as the means and standard deviations for continuous variables and as frequencies and percentages for categorical variables. The incidence of AKI will be compared between the two groups using a *χ*^2^ test. The differences in changes in SCr levels and in the eGFR will be analyzed using Student’s *t*-test or the Mann-Whitney *U* test. A value of *P* < 0.05 will be considered statistically significant. All analyses will be performed using SPSS Statistics software V21.0 (IBM Corporation, Armonk, NY, USA).

### Ethical approval

The study will be performed in accordance with the Declaration of Helsinki, as amended by the 64th World Medical Association General Assembly in 2013. All of the participants will provide written, informed consent stating that participation is voluntary and can be withdrawn at any time. This study was approved by the Institutional Review Board of the Seoul National University Bundang Hospital (B-1408/264-002). The trial protocol has been registered at http://www.clinicaltrials.gov (NCT02250872).

## Discussion

Cisplatin is an effective anti-tumor drug whose clinical application is limited by its nephrotoxicity [1, 8]. Conventional renoprotective measures in patients receiving cisplatin are not satisfactory. Many studies have investigated the effects of protective strategies targeting the molecular mechanisms of cisplatin toxicity. Although encouraging results have been found in *in vitro* and *in vivo* models, a lack of positive data in clinical trials has prevented the effective clinical application of these strategies [1, 15, 16]. DPP4 inhibitors, which are anti-diabetic drugs, have been shown to protect various organs from injuries [19–29]. This trial is the first to investigate whether a DPP4 inhibitor protects the kidney from cisplatin-induced injury in humans.

Previous studies have reported that cisplatin-induced AKI occurred in 6–35 % of patients within 7 days after a single dose of cisplatin [1, 5, 6, 31] and resolved within 15 days [7]. The incidence of AKI in patients treated with a single dose of cisplatin was 14.4 % at our institution. The average follow-up time was 17 days. However, the incidence of AKI would be higher than 14.4 % if we considered renal function within 7 days after cisplatin treatment; we estimate that the incidence of AKI would be approximately 25 %. We tentatively suggest that treatment with a DPP4 inhibitor could result in a 60 % reduction in the risk of AKI. The statistical power may be compromised if sample size is calculated using a one-sided level instead of a two-sided level. However, this trial is a phase II exploratory research, not a phase III or IV study on the possibility of direct clinical application. In addition, from the previous study [29], it is expected that DPP4 inhibitors will ameliorate rather than have no impact on or exacerbate cisplatin-induced AKI. It is not feasible to enroll more participants in our institution even though this study will be extended. Nevertheless, based on our assumptions, the sample size required for this study is larger than that of previous clinical studies of cisplatin-induced nephrotoxicity [15, 34–38].

We have considered the potential interactions between gemigliptin and cisplatin from the beginning of this study. First, cisplatin has no effect on hepatic cytochrome P450 3A4 (CYP3A4) in human liver microsomes [39], while gemigliptin is largely metabolized by CYP3A4 [40]. Second, cisplatin is mainly excreted by the kidney, and its nephrotoxicity is mediated via organic cation transporter 2 (OCT2) in the kidney [1, 41]. Gemigliptin and its active metabolite, LC15-0636, are very weak inhibitors of OCT2 in cultured xenopus oocytes. In terms of metabolic pathways and the renal transport system, cisplatin and gemigliptin never interact. Therefore, the renoprotective effect of gemigliptin could be investigated without affecting the anti-tumor activity of cisplatin.

The dose of gemigliptin in this study is two times higher than the dose used in the treatment of diabetes. Based on previous studies [29, 42], we determined that this dose was necessary to inhibit DPP4 activity by more than 80 % during cisplatin exposure.

DPP4 inhibition has not caused hypoglycemia in studies of healthy volunteers [43, 44]. Because the effects of GLP-1 on insulin secretion are glucose-dependent, the risk of hypoglycemia associated with DPP4 inhibitor treatment is low [45]. Nonetheless, we will exclude the participants with diabetes from this study. Therefore, the risk of hypoglycemia due to gemigliptin administration may be almost negligible in this trial.

A strength of this trial is that we will standardize hydration protocols before, during, and after cisplatin treatment to minimize the effects of confounding variables associated with renal outcomes. Cisplatin dosage per body surface area and the presence of diabetes have been reported to be the risk factors for moderate and severe cisplatin-induced AKI [31]. Another strength of this study is that randomization will be stratified on the basis of cisplatin dosage. Furthermore, participants with diabetes will be excluded from this study.

A small number of trials have investigated the clinical use of DPP4 inhibitors in the treatment of conditions other than diabetes. Treatments with DPP4 inhibitors have been investigated for their ability to enhance engraftment following umbilical cord blood transplantation in adults with hematologic malignancies [46], to improve endothelial dysfunction, and to prevent major cardiovascular events in patients with type 2 diabetes [47–49]. Again, we emphasize the originality of this trial, which will investigate the effect of a DPP4 inhibitor on cisplatin-induced AKI.

A limitation of this study is the diversity among the study participants and chemotherapeutic agents. Patients with different types of cancer receiving various anticancer drugs will be enrolled in this study. We expect that patients with gastric, biliary, head and neck, and lung cancer will be enrolled in this study. The following chemotherapeutic agents are used together with cisplatin in the treatment of these cancers: capecitabine, tegafur, docetaxel, pemetrexed, etoposide, irinotecan, and gemcitabine. All of these agents have been reported to have a low risk of AKI [50].

In summary, the present study is the first prospective, randomized controlled trial to evaluate the effect of a DPP4 inhibitor on cisplatin-induced AKI. The aim of this study is to demonstrate whether treatment with a DPP4 inhibitor, gemigliptin, can reduce the incidence of cisplatin-induced AKI in patients receiving cisplatin.

### Trial status

This trial is ongoing. Participants are currently being recruited.

## Abbreviations

AKI: Acute kidney injury
DPP4: Dipeptidyl peptidase-4
GLP-1: Glucagon-like peptide-1
NSAID: Nonsteroidal anti-inflammatory drug
SCr: Serum creatinine
eGFR: Estimated glomerular filtration rate
PP: Per protocol
ITT: Intention-to treat
CYP3A4: Cytochrome P450 3A4
OCT2: Organic cation transporter
